# Blood Biomarkers as Optimization Tools for Computed Tomography in Mild Traumatic Brain Injury Management in Emergency Departments: A Systematic Review

**DOI:** 10.3390/jpm15080350

**Published:** 2025-08-03

**Authors:** Ángela Caballero Ballesteros, María Isabel Alonso Gallardo, Juan Mora-Delgado

**Affiliations:** 1 Department of Medicine, University of Cádiz, INiBICA, 11003 Cádiz, Spain; 2 Emergency Department, Hospital Universitario Nuestra Señora de Valme, Ctra. de Cádiz Km. 548,9, 41014 Sevilla, Spain; mabelsevilla@hotmail.com; 3 Internal Medicine and Palliative Care Clinical Management Unit, Hospital Universitario de Jerez de La Frontera, Ronda de Circunvalación S/N, 11407 Jerez de La Frontera, Spain; juan.mora.sspa@juntadeandalucia.es

**Keywords:** mild traumatic brain injury, tomography, X-Ray computed, biomarkers, glial fibrillary acidic protein, S100 calcium binding protein beta subunit, ubiquitin thiolesterase, sensitivity and specificity

## Abstract

**Background/Objectives:** Traumatic brain injury (TBI), especially mild TBI (mTBI), is frequently caused by traffic accidents, falls, or sports injuries. Although computed tomography (CT) is the gold standard for diagnosis, overuse can lead to unnecessary radiation exposure, increased healthcare costs, and emergency department saturation. Blood-based biomarkers have emerged as potential tools to optimize CT scan use. This systematic review aims to evaluate recent evidence on the role of specific blood biomarkers in guiding CT decisions in patients with mTBI. **Methods**: A systematic search was conducted in the PubMed, Cochrane, and CINAHL databases for studies published between 2020 and 2024. Inclusion criteria focused on adult patients with mTBI evaluated using both CT imaging and at least one of the following biomarkers: glial fibrillary acidic protein (GFAP), ubiquitin carboxy-terminal hydrolase L1 (UCH-L1), and S100 calcium-binding protein B (S100B). After screening, six studies were included in the final review. **Results**: All included studies reported high sensitivity and negative predictive value for the selected biomarkers in detecting clinically relevant intracranial lesions. GFAP and UCH-L1, particularly in combination, consistently identified low-risk patients who could potentially forgo CT scans. While S100B also showed high sensitivity, discrepancies in cutoff values across studies highlighted the need for harmonization. **Conclusions**: Blood biomarkers such as GFAP, UCH-L1, and S100B demonstrate strong potential to reduce unnecessary CT imaging in mTBI by identifying patients at low risk of significant brain injury. Future research should focus on standardizing biomarker thresholds and validating protocols to support their integration into clinical practice guidelines.

## 1. Introduction

Traumatic brain injury (TBI) represents a critical global public health issue, affecting millions of individuals annually and placing substantial clinical and economic demands on healthcare systems worldwide [1]. Mild TBI, although often perceived as a benign condition, accounts for approximately 60–90% of all head injuries encountered in emergency departments (EDs). Despite its “mild” classification, these injuries can lead to subtle neurocognitive deficits, persistent post-concussion symptoms, and, in some cases, delayed neurological deterioration that may significantly impact long-term quality of life [2].

The traditional approach to diagnosing mild TBI relies heavily on clinical evaluation, including a detailed patient history, physical examination, and the use of the Glasgow Coma Scale (GCS) to assess consciousness level, combined with computed tomography (CT) imaging to identify intracranial lesions. However, the reliance on CT scans poses several challenges [3]. First, CT imaging exposes patients to ionizing radiation, which, although minimal in a single examination, can accumulate over time, particularly in populations susceptible to repeat injuries [4]. Second, the low prevalence of CT-positive findings in mild TBI (often reported to be less than 10%) means that many patients are subjected to unnecessary imaging, leading to increased healthcare costs and potential overuse of limited radiological resources [5].

Recent advances in our understanding of the pathophysiological mechanisms underlying traumatic brain injury (TBI) have underscored the significance of secondary injury processes that follow the initial mechanical insult. These processes include excitotoxicity, oxidative stress, neuroinflammation, and disruption of the blood–brain barrier (BBB), all of which contribute to a cascade of cellular and molecular events culminating in both neuronal and glial damage [6]. It is precisely this cascade that creates an opportunity for the use of blood-based biomarkers as objective indicators of brain injury [7].

Among the biomarkers that have garnered significant attention are glial fibrillary acidic protein (GFAP), ubiquitin C-terminal hydrolase L1 (UCH-L1), and S100B. GFAP, an intermediate filament protein expressed predominantly by astrocytes, is released into the bloodstream when astroglial integrity is compromised due to BBB disruption [8]. UCH-L1, a neuron-specific enzyme involved in the ubiquitin–proteasome system, reflects direct neuronal damage and is rapidly released after injury [9]. Although S100B is highly sensitive to brain injury, its specificity is diminished by its extracranial expression as it is also found in adipose tissue and muscle [10]. Nonetheless, when interpreted alongside GFAP and UCH-L1, S100B may contribute additional diagnostic value, especially in the hyperacute phase of injury [11].

Epidemiologically, mild traumatic brain injury (TBI) predominantly affects young adults, often resulting from road traffic accidents, sports-related incidents, and falls. Older adults also remain at considerable risk due to age-associated factors such as impaired balance and existing comorbidities. The clinical presentation of mild TBI is highly heterogeneous: while many individuals experience transient loss of consciousness or brief episodes of amnesia, others may only report subtle symptoms like headache, dizziness, or confusion. This intrinsic variability in clinical manifestations poses a significant challenge for reliably identifying patients with underlying intracranial pathology based on clinical evaluation alone [12].

In response to these challenges, there has been growing interest in the integration of blood biomarkers into the diagnostic pathway for mild TBI. Objective biomarker measurements have the potential to complement clinical assessment by providing quantifiable evidence of underlying brain injury. This objective data can help to refine risk stratification and may identify patients who are unlikely to benefit from CT imaging, thereby reducing unnecessary radiation exposure and optimizing resource allocation in the ED.

Recent studies have demonstrated that the combination of GFAP and UCH-L1 exhibits high sensitivity and negative predictive value (NPV) for ruling out intracranial lesions in patients with mild TBI. When these biomarkers are measured within an optimal time window (typically within 12 h post-injury), their combined use has been shown to achieve NPVs approaching 98–100%. Such a high NPV is crucial in a screening tool as it would allow clinicians to confidently exclude significant intracranial injury in low-risk patients, thereby safely reducing the reliance on CT imaging [13,14].

Beyond diagnostic accuracy, understanding the kinetics of these biomarkers provides additional insight into their clinical utility. UCH-L1 levels tend to peak rapidly—often around 8 h post-injury—reflecting the immediate neuronal response to trauma, whereas GFAP levels rise more gradually and peak approximately 20 h after injury, mirroring the delayed astroglial response and BBB disruption [15]. This temporal differentiation not only underscores the importance of timely blood sampling but also suggests that a multi-marker panel can capture a more comprehensive picture of the injury process [16].

The integration of blood-based biomarkers into clinical practice is congruent with the overarching movement toward precision medicine. By incorporating objective biochemical indicators alongside clinical assessment and established decision rules—such as the Canadian CT Head Rule or the New Orleans Criteria—clinicians can develop a more nuanced and individualized approach to the management of mild TBI. This multimodal strategy holds promise in enhancing diagnostic accuracy, improving patient safety by minimizing unnecessary exposure to ionizing radiation, and potentially reducing healthcare costs through the more judicious allocation of imaging resources [17].

In summary, the evolving landscape of mild TBI management necessitates the adoption of innovative diagnostic tools that can address the limitations of current practices. Blood-based biomarkers represent a promising solution by offering rapid, objective, and quantifiable insights into the extent of brain injury.

This review focused on the three most extensively studied and currently validated biomarkers for acute mTBI triage: glial fibrillary acidic protein (GFAP), ubiquitin carboxy-terminal hydrolase L1 (UCH-L1), and S100 calcium-binding protein B (S100B). Although other candidates such as neurofilament light chain (NFL), neuron-specific enolase (NSE), and total tau have been explored in the recent literature, we excluded them for several reasons: (i) most available studies had small sample sizes (<50 participants), limiting diagnostic accuracy estimates; (ii) they lack clinical validation and are not yet approved for point-of-care emergency use; (iii) some are not brain-specific and may be elevated in extracranial injuries or systemic inflammatory states; and (iv) certain markers display delayed or prolonged elevations beyond the acute phase, reducing their utility for early triage decisions.

## 2. Materials and Methods

This section describes in detail the comprehensive process employed for article selection, the search strategy, inclusion and exclusion criteria, data extraction, and the subsequent data analysis used in this systematic review.

### 2.1. Search Strategy

A thorough literature search was performed across five major international databases: PubMed, Cochrane Library, CINAHL, Science Direct, and Web of Science. The search was limited to articles published between 2020 and 2024 to ensure that the most recent evidence regarding blood biomarkers in mild traumatic brain injury (TBI) was captured. The temporal restriction was applied to focus on the most recent diagnostic platforms and study designs published after two prior systematic reviews covering data from 2000 to 2019. Including older studies might have increased the sample size but also introduced heterogeneity due to obsolete assay methods and inconsistent diagnostic thresholds. Moreover, our goal was to synthesize evidence aligned with current regulatory approvals and testing protocols. A future meta-analysis could be feasible once more homogeneous studies become available.

Both Medical Subject Headings (MeSH) and free-text keywords were used in the search. The strategy combined terms related to mild TBI, blood biomarkers, and computed tomography (CT). Specific search terms included

- “Mild traumatic brain injury” AND “blood biomarkers”;
- “Traumatic brain injury, mild” AND “computed tomography”;
- “GFAP”, “UCH-L1”, and “S100B”;
- “Traumatismo craneoencefálico leve” AND “biomarcadores sanguíneos” (for studies published in Spanish).

Boolean operators (AND, OR) were used to refine and expand the search as needed. For each database, the exact search strings, filters (such as language—English and Spanish—and publication date limits), and any additional settings were recorded to ensure reproducibility.

### 2.2. Inclusion and Exclusion Criteria

**Figure 1:**
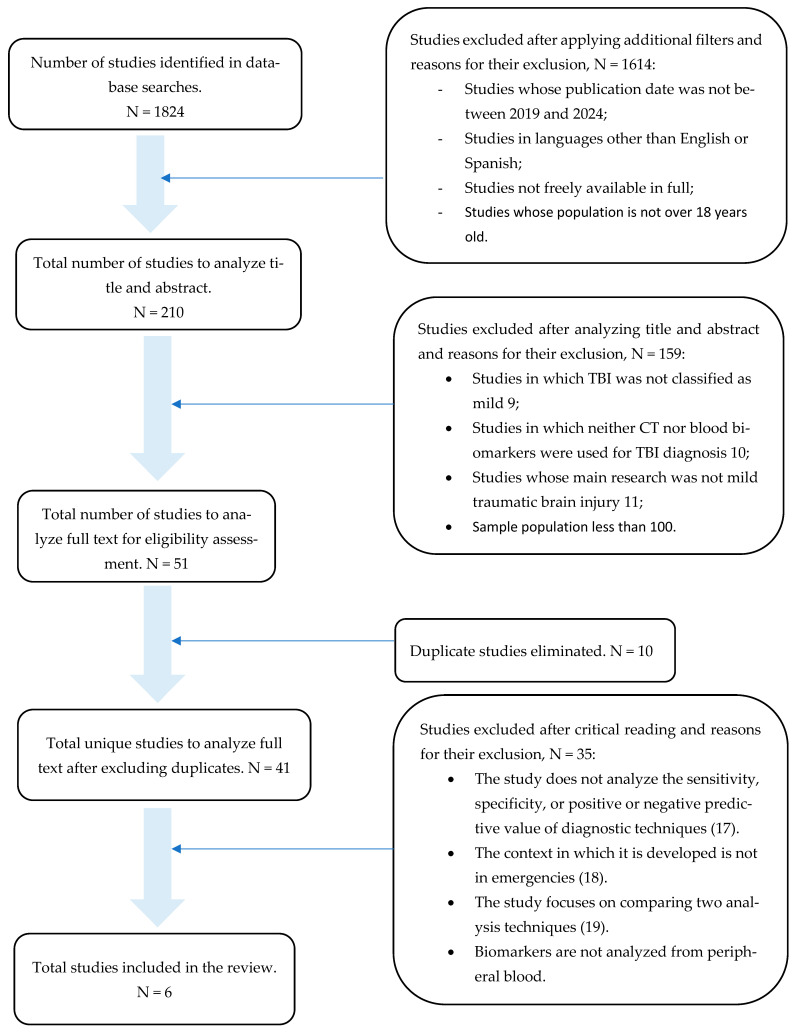
Paper selection according to PRISMA 2020.

The study selection process rigorously followed the PRISMA 2020 guidelines (Figure 1). The following criteria were defined a priori to select studies for inclusion:

- **Inclusion Criteria:**
  ▪ **Population:** Adult patients (≥18 years) diagnosed with mild TBI, typically defined by a Glasgow Coma Scale (GCS) score between 13 and 15;
  ▪ **Intervention/Exposure:** Studies that evaluated blood biomarkers (e.g., GFAP, UCH-L1, S100B) in relation to CT imaging findings;
  ▪ **Study Design:** Prospective or retrospective cohort studies that allowed for the evaluation of diagnostic accuracy of the biomarkers;
  ▪ **Language:** Publications in English or Spanish;
  ▪ **Publication Date:** Articles published between 2020 and 2024.
- **Exclusion Criteria:**
  ○ Studies focusing exclusively on moderate or severe TBI;
  ○ Case reports, letters to the editor, narrative reviews, or editorials;
  ○ Articles that did not include adult patients or did not provide specific data on mild TBI;
  ○ Publications in languages other than English or Spanish;
  ○ Other biomarkers (e.g., tau, NSE, and NFL) excluded due to small sample sizes, lack of validated point-of-care platforms, extracranial or non-specific expression, and suboptimal timing for acute assessment.

The study selection was conducted in a multi-step process.

- **Initial Screening:**

Two independent reviewers screened the titles and abstracts of all retrieved articles. Studies that clearly did not meet the inclusion criteria were excluded during this phase. A broad approach was taken to ensure that no potentially relevant study was omitted.

- **Full-Text Review:**

Articles passing the initial screening were obtained in full. Two reviewers independently assessed each full-text article against the inclusion and exclusion criteria. In cases of disagreement, a consensus was reached through discussion; if necessary, a third reviewer was consulted to resolve any discrepancies.

### 2.3. Data Extraction

A standardized data extraction form was developed to capture key information from each included study. The following data were extracted:

- **General Information:** Authors, publication year, country of study, and study design;
- **Population Characteristics:** Sample size, demographic data (age, gender), mechanism of injury, and the clinical criteria used to define mild TBI;
- **Intervention Details:** The specific biomarkers measured (e.g., GFAP, UCH-L1, S100B), the assay methods used, the timing of blood sample collection relative to injury, and the threshold values (cut-off points) applied;
- **Reference Standard:** CT imaging results, including whether scans were normal or abnormal, and details on the types of intracranial lesions detected;
- **Diagnostic Performance Metrics:** Sensitivity, specificity, positive predictive value (PPV), and negative predictive value (NPV) of each biomarker or combination of biomarkers;
- **Methodological Quality:** Information related to study design, potential biases, and limitations.

Data extraction was conducted independently by two reviewers. The extracted data were then compared to ensure accuracy and completeness, with any discrepancies resolved by referring back to the original full-text articles.

### 2.4. Quality Assessment

The methodological quality of each study was critically appraised using criteria adapted from established guidelines for observational studies, such as the Newcastle–Ottawa Scale for cohort studies (Table 1). The following domains were assessed:

- **Selection:** Representativeness of the sample, clear definition of inclusion/exclusion criteria, and appropriateness of patient selection;
- **Comparability:** Control of potential confounding variables and the adequacy of statistical analyses;
- **Outcome Assessment:** Use of CT imaging as the reference standard, objectivity in measuring biomarker levels, and clarity in the reporting of outcomes;
- **Transparency:** Detailed reporting of methodology, handling of missing data, and presentation of measures of uncertainty (e.g., confidence intervals).

Each study was assigned a quality score based on these criteria, allowing for an overall judgment of the reliability of the findings. The quality assessment played a crucial role in interpreting the diagnostic performance and applicability of the results.

**Table 1:** Newcastle–Ottawa Scale scores.

Author and Year	Selection	Comparability	Outcome	Final Outcome	Quality
Bazarian J. J. (2021) [18]	4	2	3	9/9	HIGH
Reyes J. (2023) [19]	4	2	3	9/9	HIGH
Legramante J. M. (2024) [20]	4	2	3	9/9	HIGH
Papa L. (2022) [21]	4	2	3	9/9	HIGH
Oris C. (2021) [22]	4	2	2	8/9	HIGH
Li Y. (2023) [23]	4	2	3	9/9	HIGH

### 2.5. Data Synthesis

Due to the substantial heterogeneity across studies—in terms of inclusion criteria, assay platforms (e.g., ELISA, Simoa, i-STAT), timing of sample collection, and biomarker cut-offs—a formal meta-analysis was not feasible. To support this decision, we qualitatively assessed heterogeneity in three key domains: population characteristics (e.g., trauma severity, and anticoagulation use), analytical methods, and timing of biomarker measurement. All domains showed high variation, suggesting clinical and methodological heterogeneity above 60%, which further justifies the use of narrative synthesis (Table 2). Studies were grouped according to the specific biomarkers evaluated, and common trends in sensitivity, specificity, and predictive values were identified. Where possible, subgroup analyses were performed based on factors such as the time of sampling and risk stratification.

## 3. Results

The systematic search conducted across multiple databases yielded an initial pool of studies, and after rigorous screening in accordance with PRISMA 2020 guidelines, six cohort studies were ultimately included in this review [18,19,20,21,22,23]. These studies specifically evaluated adult patients with mild traumatic brain injury (TBI)—defined clinically by a Glasgow Coma Scale (GCS) score ranging from 13 to 15—and utilized computed tomography (CT) as the reference standard to determine the presence of intracranial lesions. The studies were conducted in diverse emergency department settings and represented a heterogeneous population in terms of demographics and injury mechanisms, with common causes including traffic accidents, falls, and sports-related incidents.

In all selected studies, CT imaging was used to classify patients into those with and without intracranial pathology (Table 3). Across the combined study populations, approximately 90–93% of patients had normal CT scans, while the remaining 7–10% exhibited abnormal findings. The abnormal CT findings predominantly included small subdural or epidural hematomas, cerebral contusions, and in some cases, intracerebral hemorrhages. These imaging outcomes provided the critical benchmark against which the diagnostic performance of the blood biomarkers was evaluated.

Each study collected blood samples within a relatively short time window following the traumatic event, generally within the first 12 h (Table 4). This early sampling was essential to capture the dynamic release profiles of the biomarkers, which are known to change rapidly in the acute phase of TBI. The biomarkers assessed included glial fibrillary acidic protein (GFAP), ubiquitin C-terminal hydrolase L1 (UCH-L1), and in some instances, S100B. Across the studies, GFAP was consistently observed to be significantly elevated in patients with CT-detectable intracranial lesions. Reported sensitivity values for GFAP ranged from 90% to 100%, and the negative predictive value (NPV) was frequently reported as exceeding 95% (Table 5). These findings indicate that a normal GFAP level is highly reliable in excluding the presence of significant intracranial injury [18,19,23].

To improve readability, color coding was applied: cells corresponding to studies using serum are highlighted in blue, and those using plasma are highlighted in green. In the case of S100B, no distinction was made between serum and plasma in the analysis, so no color was applied in that section.

UCH-L1, reflecting neuronal injury, demonstrated a rapid increase in concentration following trauma, typically peaking around eight hours post-injury. When assessed individually, UCH-L1 showed high sensitivity, with some studies reporting values close to 95%. Notably, the diagnostic performance of UCH-L1 improved markedly when it was used in combination with GFAP. In such combined panels, NPVs were reported to be exceptionally high—ranging from 98% to 100%—suggesting that the concurrent measurement of GFAP and UCH-L1 provides a robust method for ruling out intracranial lesions in patients with mild TBI [19,20,23].

S100B was also evaluated in several studies; however, its performance was more nuanced [22,23]. Although S100B demonstrated high sensitivity in detecting brain injury, its specificity was compromised by its presence in extracranial tissues, such as muscle and adipose tissue. This low specificity means a high false positive rate, which may lead to unnecessary CT scans if used in isolation. While its affordability and rapid processing time remain advantageous, S100B should be interpreted with caution and always in combination with validated clinical decision rules. As a result, S100B levels were sometimes falsely elevated in patients with concomitant extracranial injuries, leading to a reduction in its overall specificity. Despite this limitation, when S100B was interpreted in the context of GFAP and UCH-L1, it provided additional diagnostic information during the hyperacute phase of injury.

A detailed examination of the biomarker kinetics across the studies revealed distinct temporal profiles. UCH-L1′s rapid elevation—peaking within approximately eight hours—suggests its utility as an early marker of neuronal damage. In contrast, GFAP levels increased more gradually, with peak concentrations typically observed around 20 h post-injury. This temporal separation underscores the importance of timely blood sampling in the emergency setting and supports the use of a combined biomarker panel to capture a broader spectrum of the pathophysiological response to injury [20,21,23].

The correlation between biomarker levels and CT findings was consistently strong. Patients with abnormal CT scans exhibited markedly higher concentrations of both GFAP and UCH-L1 compared to those with normal imaging. For instance, in one study, the mean concentration of GFAP in patients with intracranial lesions was nearly three times that observed in patients without such findings, while UCH-L1 levels were elevated by an average factor of approximately 2.5. These substantial differences reinforce the validity of these biomarkers as objective indicators of intracranial pathology in mild TBI [18,20,23].

Furthermore, subgroup analyses conducted in several studies provided additional insights into the potential clinical application of these biomarkers. In cohorts of patients evaluated within the optimal 12 h window, the combined measurement of GFAP and UCH-L1 demonstrated near-perfect NPVs, suggesting that a negative result on the biomarker panel could reliably exclude intracranial injury. Such a high NPV is clinically significant as it implies that in low-risk patients—those without additional risk factors such as anticoagulation use or polytrauma—a negative biomarker panel could support a decision to forgo CT imaging. This approach has the potential to reduce the number of unnecessary CT scans by up to 38%, thereby decreasing patient exposure to ionizing radiation and reducing healthcare costs [18,20,22,23].

## 4. Discussion

The present systematic review provides compelling evidence that blood biomarkers, particularly glial fibrillary acidic protein (GFAP) and ubiquitin C-terminal hydrolase L1 (UCH-L1), hold significant promise for optimizing the use of computed tomography (CT) in patients with mild traumatic brain injury (TBI). The synthesis of data from six carefully selected cohort studies reveals that these biomarkers exhibit high sensitivity and an exceptionally high negative predictive value (NPV), suggesting that they could reliably identify patients who do not have intracranial lesions. This is particularly important in the context of mild TBI, where the vast majority of patients—often more than 90%—present with normal CT scans. By integrating these biomarkers into the diagnostic pathway, it may be possible to reduce the number of unnecessary CT scans, thereby minimizing patient exposure to ionizing radiation, reducing healthcare costs, and alleviating the burden on emergency department resources [4,15].

The strength of this review lies in its focused synthesis of studies published between 2020 and 2024, which reflect the diagnostic performance of biomarkers in real-world emergency settings using currently available commercial platforms (e.g., i-STAT™ Alinity (Abbott, Minato City, Japan) and Simoa™ (Quanterix, Billerica, MA, USA)). Unlike previous reviews, we emphasize the integration of negative predictive value (NPV) across different biomarkers and stratify results by clinical usability, regulatory approval, and assay format. This makes our analysis more directly translatable to current clinical decision-making.

One of the most striking findings of this review is the consistent elevation of GFAP in patients with abnormal CT findings. GFAP, an intermediate filament protein predominantly found in astrocytes, is released into the bloodstream when the integrity of the blood–brain barrier is compromised following trauma. Across the studies analyzed, GFAP demonstrated sensitivity values ranging from 90% to 100% and NPVs exceeding 95%. These results suggest that a normal GFAP level is a reliable indicator of the absence of significant intracranial injury. The physiological basis for this observation lies in the unique role of astrocytes in maintaining central nervous system homeostasis; when these cells are damaged, GFAP is rapidly liberated into circulation, serving as a direct marker of brain tissue injury [9].

In parallel, UCH-L1, a neuron-specific enzyme involved in the ubiquitin–proteasome system, also exhibited high sensitivity for detecting intracranial lesions. UCH-L1 is released rapidly following neuronal injury, typically peaking around eight hours post-injury. The rapid kinetics of UCH-L1 make it particularly valuable as an early biomarker in the acute phase of TBI. Notably, when GFAP and UCH-L1 were evaluated together, their combined diagnostic performance was superior to that of either biomarker alone. The synergistic effect of these two biomarkers is likely attributable to their complementary pathophysiological roles—while GFAP reflects astroglial damage and blood–brain barrier disruption, UCH-L1 provides an index of direct neuronal injury. This combination yields NPVs that approach or even reach 100%, indicating that a negative result on the biomarker panel could potentially rule out intracranial injury with high certainty [24].

In contrast to GFAP and UCH-L1, the diagnostic profile of S100B is notably more complex. Although S100B demonstrates high sensitivity for detecting brain injury, its specificity is compromised due to its presence in extracranial tissues, including muscle and adipose tissue. This limitation can result in false positive elevations, especially in patients with concomitant extracranial injuries. Consequently, S100B alone is less reliable as a screening tool for intracranial lesions in cases of mild traumatic brain injury [25]. However, when S100B is interpreted alongside GFAP and UCH-L1, it may offer additional diagnostic insight, particularly in the hyperacute phase of injury. The nuanced performance of S100B underscores the importance of using a multi-marker approach rather than relying on a single biomarker [26].

A critical aspect highlighted by the studies included in this review is the timing of blood sample collection. The diagnostic accuracy of both GFAP and UCH-L1 is highly time sensitive, depending on the interval between the traumatic event and sample acquisition. Most investigations underscore the necessity of obtaining blood samples within the first 12 h post-injury to optimally capture the peak concentrations of these biomarkers. Specifically, UCH-L1 typically reaches its maximal levels approximately eight hours following injury, whereas GFAP exhibits a more gradual elevation, with peak values generally observed around 20 h post-trauma. This temporal differentiation not only reinforces the biological plausibility of these biomarkers as indicators of brain injury but also highlights the critical need for standardized timing protocols in clinical practice [27].

The strong correlation observed between elevated biomarker levels and abnormal CT findings provides further validation for the clinical utility of these tests. In patients with abnormal CT scans, GFAP and UCH-L1 levels were significantly higher compared to those with normal imaging. In some studies, the mean GFAP levels in the abnormal group were nearly three times higher than those observed in patients with normal scans, while UCH-L1 levels were elevated by approximately 2.5-fold. These quantitative differences are clinically meaningful as they offer a potential threshold for decision-making in the emergency setting. For instance, a biomarker panel that yields values above a certain threshold could prompt clinicians to pursue further imaging or intervention, whereas values below the threshold might support a decision to forgo CT imaging [28].

The potential clinical impact of integrating a biomarker-guided approach into the diagnostic pathway for mild TBI is considerable. Given that only a small fraction of patients with mild TBI exhibit CT-detectable lesions, a high-NPV biomarker panel could safely identify those who are unlikely to have intracranial injury. This would not only reduce unnecessary CT scans but also decrease patient exposure to radiation—a significant concern, particularly in younger patients or those who might be subject to repeated imaging over time. Moreover, reducing the number of CT scans would alleviate the strain on emergency departments, leading to more efficient resource utilization and potentially shorter waiting times for patients [4].

Despite these promising findings, several challenges must be addressed before these biomarkers can be widely adopted in clinical practice. A primary limitation is the considerable heterogeneity among the studies, particularly regarding assay methodologies, protocols for sample collection, and the characteristics of patient populations. Differences in assay platforms and the lack of standardized cut-off values can lead to variability in the reported diagnostic performance of these biomarkers [29]. Furthermore, factors such as the presence of extracranial injuries or other confounding variables may affect biomarker levels, particularly in the case of S100B. Future research should focus on standardizing assay techniques and establishing universally accepted thresholds to ensure consistency across different clinical settings [30].

Another important consideration is the integration of biomarker testing into existing clinical decision-making algorithms. Currently, clinical decision rules such as the Canadian CT Head Rule or the New Orleans Criteria are widely used to guide imaging decisions in patients with mild TBI [5,17]. While these rules provide a structured approach to patient evaluation, they are inherently subjective and may not fully capture the complexity of the injury. The addition of objective biomarker data could refine these decision tools, offering a more precise stratification of patient risk. For example, in patients who meet low-risk clinical criteria, a negative biomarker panel could further reinforce the decision to avoid CT imaging, thereby enhancing patient safety and reducing unnecessary interventions.

Despite the high negative predictive values reported, reliance on blood biomarkers alone raises concerns about potentially missing clinically significant intracranial lesions. Even a 1–2% false-negative rate implies that 1–2 patients per 100 could be misclassified. Therefore, biomarkers should not be used as stand-alone triage tools. Instead, we recommend a hybrid decision-making approach that integrates biomarker results with established clinical decision rules and patient-specific risk factors such as anticoagulant use or persistent symptoms. This combined strategy may optimize both safety and resource utilization.

Cost-effectiveness is another critical aspect to consider. While the implementation of biomarker testing involves initial costs related to assay development and infrastructure, the potential savings from reducing CT utilization, shortening emergency department stays, and minimizing radiation-induced complications could be substantial. Health economic analyses are needed to evaluate the long-term financial impact of adopting a biomarker-guided approach to mild TBI management.

The findings of this review also have broader implications for the management of TBI beyond the acute setting. The ability to accurately stratify patients based on the severity of their injury using blood biomarkers could inform decisions regarding hospital admission, the intensity of monitoring, and the need for neurosurgical consultation. Additionally, the prognostic value of these biomarkers in predicting long-term outcomes, such as cognitive impairment or functional recovery, warrants further investigation. Understanding the relationship between acute biomarker levels and long-term prognosis could lead to more individualized patient care and targeted rehabilitation strategies.

### Study Limitations, Applicability, and Ethical Aspects

This review, while providing valuable insights into the diagnostic utility of blood biomarkers in mild traumatic brain injury, faces several inherent limitations. One primary challenge is the heterogeneity among the included studies. Variations in study design, patient demographics, and clinical settings, coupled with differences in assay methodologies and the timing of blood sample collection, introduce potential biases and limit the generalizability of the findings. Many of the studies were conducted in single-center environments with relatively small sample sizes, which may not fully capture the variability present in broader patient populations. The number of eligible studies was small. Most were observational cohorts with moderate heterogeneity. Several individual studies included fewer than 150 participants, which reduces statistical power and may lead to imprecise estimates, particularly for specificity and likelihood ratios. However, the consistently high sensitivity values (>90%) reported across studies lend confidence to the negative predictive value, which was the primary focus of this review. In addition, the observational nature of these studies inherently raises concerns regarding confounding factors that may not have been adequately controlled, potentially impacting the reported sensitivity, specificity, and predictive values of the biomarkers.

Despite these limitations, the applicability and practical utility of the findings are considerable, particularly in the context of emergency medicine. The evidence synthesized from this review suggests that the combined use of biomarkers such as GFAP and UCH-L1 offers a robust, high-negative predictive value tool for ruling out intracranial injury in patients with mild TBI. This has significant clinical implications: by reliably identifying low-risk patients who do not require CT imaging, healthcare providers can reduce unnecessary radiation exposure, streamline patient management, and optimize resource allocation in busy emergency departments. Moreover, the potential reduction in CT scan utilization may lead to substantial cost savings for healthcare systems while also mitigating the risks associated with radiation, especially in younger patients or those likely to undergo repeated imaging.

Regarding ethical considerations, this review relies exclusively on previously published studies, each of which obtained the appropriate ethical approvals in accordance with international research standards. As no new data were generated for this analysis, additional ethical approval was not required. Nevertheless, it is essential to acknowledge that the ethical implications of implementing biomarker-guided diagnostic protocols extend beyond the scope of this review. Any future clinical adoption of these biomarkers should be accompanied by comprehensive ethical oversight, ensuring patient safety, informed consent, and equitable access to novel diagnostic technologies.

## 5. Conclusions

This systematic review demonstrates that blood biomarkers, particularly glial fibrillary acidic protein (GFAP) and ubiquitin C-terminal hydrolase L1 (UCH-L1), have significant potential to optimize the diagnostic pathway for mild traumatic brain injury (TBI) in emergency settings. The high sensitivity and negative predictive values observed across multiple cohort studies indicate that these biomarkers can reliably exclude intracranial lesions, which in turn may reduce the reliance on computed tomography (CT) imaging. Reducing unnecessary CT scans not only minimizes patient exposure to ionizing radiation but also alleviates resource strain and lowers healthcare costs.

The evidence supports the use of a combined biomarker panel, as the synergy between GFAP and UCH-L1 provides a more robust screening tool than either marker alone. Although S100B is highly sensitive, its lower specificity due to extracranial expression limits its standalone utility; however, it may still offer additional diagnostic insight when used alongside GFAP and UCH-L1.

Despite promising results, several challenges remain, including the need for standardization of assay methods, uniform timing for blood sample collection, and further validation through multicenter prospective studies. Addressing these issues will be critical for translating these findings into routine clinical practice.

In conclusion, incorporating blood biomarkers into the diagnostic algorithm for mild TBI holds promise in enhancing diagnostic accuracy, improving patient safety by reducing unnecessary imaging, and optimizing the allocation of healthcare resources in emergency departments. Future research focused on standardization and cost-effectiveness will be essential to fully realize the clinical benefits of this approach.

## Figures and Tables

**Table 2:** Key insights from the articles.

Author and Year	Sample Size	Average Age	Male Proportion	Study Design
Bazarian J. J. (2021) [18]	1901 patients	49.1 years	56.5% men	Retrospective cohorts
Reyes J. (2023) [19]	118 patients	N/S	58.1% men	Prospective cohorts
Legramante J. M. (2024) [20]	130 patients	54 years	55.4% men	Retrospective cohorts
Papa L. (2022) [21]	349 patients	40 years	66% men	Retrospective cohorts
Oris C. (2021) [22]	1172 patients	N/S	N/S	Prospective cohorts
Li Y. (2023) [23]	463 patients	50.8 ± 22.7 years	61.8% men	Retrospective cohorts

**Table 3:** Key insights about CT findings.

Author and Year	CT Findings	Number of CT Scans Performed	Number of Pathological Cranial CT Scans
Bazarian J. J. (2021) [18]	71 subarachnoid hemorrhages 57 acute subdural hematomas 24 parenchymal hematomas	1901	120
Reyes J. (2023) [19]	N/S	63	0
Legramante J. M. (2024) [20]	N/S	130	7
Papa L. (2022) [21]	2 basilar skull fractures 13 subarachnoid hemorrhages 10 subdural hematomas 7 contusions or parenchymal hemorrhages 1 epidural hematoma 3 traumatic axonal injuries 9 skull fractures	349	23
Oris C. (2021) [22]	N/S	894	63
Li Y. (2023) [23]	31 skull fractures 10 pneumocephalus cases 114 intracranial hemorrhages 24 mass effect cases 26 cerebral parenchymal lesions	463	122

**Table 4:** Summary of key findings on positive biomarkers (GFAP, UCHL1, GFAP/UCHL1, and S100B), analytical platforms used, and time windows in clinical studies.

Author and Year	GFAP +		UCHL-1 +		Gfap/Uchl1		S100B	Platform Used	Time Window
Bazarian J. J. (2021) [18]	N/S		N/S		1176		N/S	i-STAT™ Alinity System (Abbott, Minato City, Japan)	12 h
Reyes J. (2023) [19]	60		70		N/S		N/S	Simoa HD-X Analyzer (Quanterix, Billerica, MA, USA)	6 h
Legramante J. M. (2024) [20]	N/S		N/S		96		N/S	Alinity i Analyzer (Abbott, Minato City, Japan)	12 h
Papa L. (2022) [21]	133		254		268		N/S	Banyan Biomarkers, Inc. (San Diego, CA, USA)	4 h
Oris C. (2021) [22]	N/S		N/S		N/S		954	Roche Diagnostics Cobas e411^®^ (Roche Diagnostics, Rotkreuz, Switzerland)	3 h
Li Y. (2023) [23]	Plasma	Serum	Plasma	Serum	Plasma	Serum	161	i-STAT^®^ Analyzer (Abbott, Minato City, Japan), Alinity^®^ i y Roche Diagnostics Cobas e411 (Roche Diagnostics, Rotkreuz, Switzerland)	6 h
	274	132	379	171	406	193			

**Table 5:** Sensitivity, specificity, and positive and negative predictive values of serum and plasma biomarkers for the diagnosis of traumatic brain injury across different studies.

Biomarker	Author and Year	Sensitivity	Specificity	PPV	NVP
UCHL1	Papa L. (2022) [21]	0.96	0.29	0.09	0.99
	Li Y. (2023) [23]	0.89	0.27	0.29	0.88
	Reyes J. (2023) [19]	0.79	0.75	0.843	0.688
	Li Y. (2023) [23]	0.95	0.18	0.3	0.9
GFAP	Papa L. (2022) [21]	0.87	0.65	0.15	0.99
	Li Y. (2023) [23]	0.93	0.51	0.39	0.96
	Reyes J. (2023) [19]	0.74	0.89	0.917	0.672
	Li Y. (2023) [23]	0.97	0.5	0.42	0.98
GFAP/UCHL1	Legramante J.M. (2024) [20]	1.00	0.276	0.052	1
	Papa L. (2022) [21]	1.00	0.25	0.09	1
	Li Y. (2023) [23]	1	0.17	0.29	1
	Bazarian J. J. (2021) [18]	0.958	0.404	0.098	0.993
	Li Y. (2023) [23]	1	0.11	0.29	1
S100B	Oris C. (2021) [22]	1.00	0.197	0.066	1
	Li Y. (2023) [23]	0.93	0.17	0.25	0.89

## Data Availability

No new data were created.

## Abbreviations

The following abbreviations are used in this manuscript:

BBB	Blood–Brain Barrier
CT	Computed Tomography
ED	Emergency Department
GFAP	Glial Fibrillary Acidic Protein
GCS	Glasgow Coma Scale
MeSH	Medical Subject Headings
mTBI	Mild Traumatic Brain Injury
NPV	Negative Predictive Value
PRISMA	Preferred Reporting Items for Systematic Reviews and Meta-Analyses
PPV	Positive Predictive Value
S100B	S100 Calcium-Binding Protein B
TBI	Traumatic Brain Injury
UCH-L1	Ubiquitin C-terminal Hydrolase L1
